# Determination of L- Ascorbic Acid in Plasma by Voltammetric Method

**Published:** 2010

**Authors:** Abdol Azim Behfar, Nafiseh Sadeghi, Behrooz Jannat, Mohammad Reza Oveisi

**Affiliations:** a Department of Food Chemistry and Medical Hydrology, Faculty of pharmacy, Ahvaz Jundishapoor of University Medical Sciences, Ahvaz, Iran.; b Drug and Food Control Department, Faculty of *P*harmacy, University Tehran of Medical Sciences.; c Food and Drug Laboratory Research Center, Food and Drug Deputy, Ministry of Health treatment and Medical Education, Tehran, Iran.

**Keywords:** Voltammetry, L-ascorbic acid determination, Gold electrode, Copper

## Abstract

Voltammetric techniques have been considered as important methods among the analytical techniques used for the identification and determination of trace concentrations of many biological molecules such as L-ascorbic acid (AA). L-ascorbic acid is an electro-active molecule, though it is difficult to determine its value directly with a majority of electrodes made of carbon and transition metals, because of electrode surface problems. The present study is based on I-E curves for AA analysis at various pH. Furthermore, the effects of the presence of other electro-active substances; such as copper, as well as the effect of the sweep rate of potential will be studied.

The present study is based on analysis of the current-voltage curves for L-ascorbic acid at varying pH and sweep rate scan values. An analysis was also carried out to measure the influence of the concentration of some electro active species. The peak height of the first oxidation wave is used for L-ascorbic acid assay.

L-ascorbic acid was determined in aqueous media by linear-scan voltammetry on a gold electrode; ranging between (1-175 μg/mL). In biologic samples, for elimination of uric acid or some sugars and effects, a significant interference of copper ions whose presence reduces the height of the L-ascorbic acid oxidation peak was used. The optimum pH and sweep rate were 3.2 and 7500mV/s, respectively. Under these conditions, the detection limit of the method was 0.3 μg/mL. Repeatability of the method based on relative standard deviation (RSD) 50, 10 and 1 μg/mL concentrations was 0.83, 2.1 and 10.3%, respectively. The calibration curve was linear over the range 1-175μg/mL (r^2 ^= 0.9977, p < 0.001).

The advantage of this method lies in the fact that the use of copper eliminates the interference of different substances such as uric acid.

## Introduction

Voltammetric techniques are based on the measurement of the current arising from oxidation or reduction on an electrode surface following the application of variable potential (1). These techniques have popularity among analytical techniques for the identification and measurement of trace amounts of organic, biological and inorganic species (2, 3). The methods include linear sweep voltammetry (LSV), cyclic voltammetry (CV), differential pulse voltammetry (DPV) (4) square-wave voltammetry (SWV) (5) anodic or cathodic stripping voltammetry (ASV & CSV) (2, 3) adsorptive stripping voltammetry (AdSV) (6) and electrochemical immunoassay (7).

Traces of biologic molecules of organic and inorganic description such as pollutants, drugs, food additives, can be measured usually after initial treating and conditioning by using certain separation and voltammetric techniques. It is possible for every species having a oxidation or reduction potential, to be determined directly within a matrix (1).

L-ascorbic acid can be found in all animal and plant species (8). Its main function is anti-oxidant property to protect textures and tissues from damages caused by free radicals and it also maintains some enzymes in a reduced form (9, 10). The mechanisms of these reactions are often unknown. However, recently, the details of the specific reaction of ascorbic acid with calcium channel (11) and neuro-transmitter receptors (12) have been discussed. The high concentrations of ascorbic acid in adrenal glands (13) and the brain (14) indicate the importance of this molecule operation in these organs (15-17).

L- ascorbic acid is an electro-active molecule, because of electrode surface problems it is difficult to be determined directly with a majority of electrodes made of carbon and transition metals (18-23). A great deal of studies about AA oxidation have been performed by using mercury drop electrode (24-26). However, There is few information concerning ascorbic acid behavior at the surface of gold electrodes, in spite of the fact that using this metal can bring about some improvements due to wide range of anodic polarizations and ease of electrical conduction (27- 29).

The present study is based on I-E curves for ascorbic acid analysis at various pH. Furthermore, the effects of the presence of other electro-active substances especially copper as well as effect of the sweep rate of potential on the shape of I-E curves and ascorbic acid determination are studied.

## Experimental

*Apparatus*

Voltammograms were prepared using a trace analyzer, model 747 (Metrohm AG Ltd., Switzerland). The voltammetric cell was made of borosilicate glass and had a working volume 5-50 mL. The cell was a three-electrode system with a saturated Ag/AgCl electrode, a platinum electrode and a gold electrode, as reference electrode, auxiliary electrode and working electrode, respectively. The working electrode contained a gold-plated disc of 2 mm diameter and was covered with teflon. 

*Reactants and solutions*

All the reagents were purchased from Merck Co. (Germany). We used extra pure ascorbic acid (≥ 99.7%) and double distilled water (≤ 3μS/cm). Buffer solutions with different pH were made from an initial mixture of CH_3_COONa (0.05M), Na_2_HPO_4_ (0.05M), Na_2_B_4_O_7_ (0.05M) and CH_3_COOH (0.05M), H_3_PO_4_ (0.05M) and H_3_BO_3_ (0.05M). 

Since ascorbic acid solutions were unstable, they were made immediately prior to any test and were added to a deoxygenated supporting electrolyte. All determinations were carried out under nitrogen atmosphere.

*Sample preparation*

Venous blood samples were obtained from the forearm vein and were transferred to with heparinated tubes. Plasma was separated by centrifugation at 3500 g for 5 min at 4 °C.

*Voltammetric determination*

10 mL of the buffer (pH = 2-12), 0.5 mL of the ascorbic acid standards or sample solutions were pipetted into the voltammetric cell. The solution was deoxygenated while being stirred for 180s by highly purified nitrogen. The stirring was stopped and the potential was scanned with a sweep rate of 5-10000 mV/s, from -100 mV to +1500 mV, 30s after the quiescent of solution. 

## Results and Discussion

Several experiments were performed to determine the optimum conditions for the best sensitivity, robustness and accuracy in trace quantities of ascorbic acid analysis. All experiments were performed in linear sweep mode using the buffer as a supporting electrolyte.

**Figure 1:**
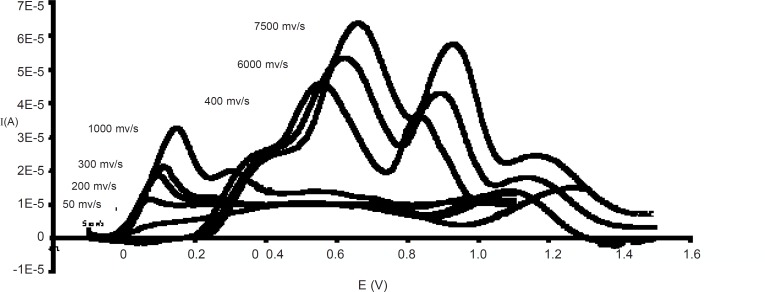
Effect of scanning rate on voltammograms at pH 3.2

*Effect of pH*

The supporting electrolyte was acetate-phosphate-borate buffer (0.05 M). Within the pH range of about 2-12, L-ascorbic acid had two oxidation waves, which the second one could be readily observed at sweep rates higher than 1000 mV/s.

The peak potential of the first wave shifted less towards anodic potentials by increasing the pH from 2 to 12, though the shape and height of this peak do not depend on the changes in pH level.

*Effect of scan rate*

By scanning rate of about 5-100 mV/s, ascorbic acid curve assumed to form a sigmoid shape. However, in the rate range of 100-1000 mV/s only one peak appeared and the second one was observable at scanning rate higher than 1000 mV/s. 

The first oxidation peak moves to more anodic potentials when the scanning rate is increased. However, increasing in the scanning rate causes the second wave to become a peak (Figure1). 

*Determination of L-ascorbic acid in plasma*

Of the two peaks, the first one was used to measure L-ascorbic acid concentrations. This experiment was carried out under an optimum pH of 3.2 and an optimum scanning rate of 7500 mV/s. 10 mL of buffer (0.05 M, pH = 3.2) was pipetted into the voltammetric cell, followed by the addition of 0.5mL of the standard or sample solution. The solution was deoxygenated for 180s by highly purified nitrogen, while the solution was stirring. The stirring was stopped and the potential was scanned from –100 mv to +1500 mv at a rate of 7500 mV/s, 30s after the solution was quiescent. 

**Figure 2:**
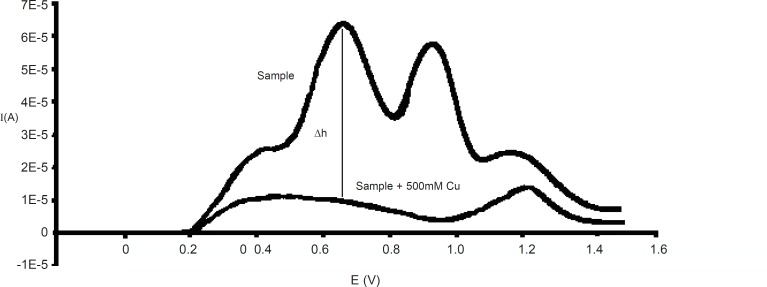
Voltammograms of L-ascorbic acid in plasma before and after of copper addition

In the biological sample studies, EDTA produces a large wave, which overlapped with the ascorbic acid wave. In the cases which blood plasma is used, it would be better to use heparin as an anti-coagulant instead of EDTA, because it may form a peak in this conditions, and so the most noticeable interference will be that of uric acid (30, 31). The uric acid peak and oxidation peak of L-ascorbic acid have overlaps because of their ΔE≤30 mV. On the other hand, another significant interference is that of copper ions whose presence reduces the height of the ascorbic acid oxidation peak (32). In fact, if determination is made immediately after the sample preparation, copper ions will not have any influence on other reactions. However, the peak height of L-ascorbic acid will reduce to zero after the addition of 500 mM copper at 37 °C for a period of one hour. Copper decreases the height of ascorbic acid peak to zero but it does not affect uric acid peak height; therefore, it can be used to eliminate the interference of uric acid and other substance with ascorbic acid. Thus, each sample or standard solution was divided into two parts which to one part was added 500 mM copper and the resulting mixture was kept at 37 °C for one hour before analysis; however the other part was analyzed immediately at room temperature without adding copper. The difference between the height peaks in the two analyses (sample without copper, sample + 500 mM copper) could be attributed to the amount of L-ascorbic acid (Figure 2). L-ascorbic acid solution in 1, 5, 10, 20, 50, 75, 100, 125, 150, 175 μg/mL concentrations were used as standards for calibration curve. 

The limit of linear response was known to be 1-175 μg/mL. The correlation coefficient of linear regression (r^2^ = 0.998) was used to determine the linear equation, which is: 

Y = (1.1×10^-7^) + (3.6×10^-7^) X

Y and X are current and concentration, respectively.

The analytical performance characteristics, which showed the validity of the method, are summarized in Table 1.

**Table 1:** The analytical performance characteristics of the method.

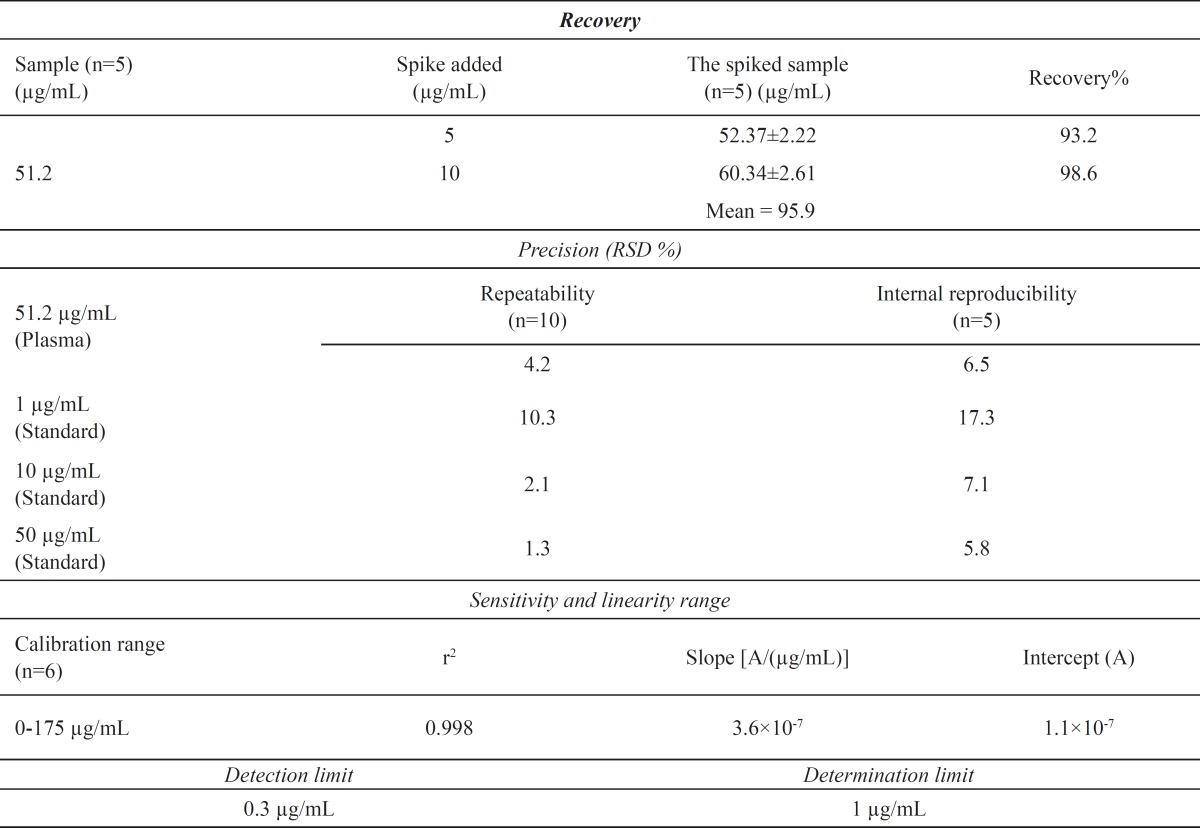

Some electrochemical studies on ascorbic acid analysis had used the modified electrods such as Zeolite-Modified Carbon-Past electrodes and Poly (*p*-toluene sulfonic acid) Modified Electrodes to eliminate the interaction of electroactive molecules. All of these methods need sampling or electrode preparation, but in our investigation, only addition of copper dissolve the problems of L-ascorbic acid measurement (33-36). Further, it was also found out that neither sugars such as dextrose, lactose, glucose nor elements such as magnesium, cobalt, chromium, calcium and manganese ions could affect the quantization even at concentrations of about 100 times greater than that of lascorbic acid. The lowest limit was determined by Sahbaz and Somer (32) that is 25-250 μg/mL. This difference in limit of determination seems to be due to the fact that base line was eliminated in our method and also to the fact that we used gold electrode instead of mercury electrode because of anodic potential limitation of the latter. 

Based on the obtained results, we can conclude that the proposed method is suitable to determine L-ascorbic acid. The method is readily and inexpensively applicable. The advantage of this method lies in the fact that it uses copper to eliminate the interference of different substances such as uric acid. 
